# Systematic review and meta-analysis of traditional Chinese medicine in the treatment of constipation-predominant irritable bowel syndrome

**DOI:** 10.1371/journal.pone.0189491

**Published:** 2017-12-18

**Authors:** Dan-yan Li, Yun-kai Dai, Yun-zhan Zhang, Meng-xin Huang, Ru-liu Li, Jia Ou-yang, Wei-jing Chen, Ling Hu

**Affiliations:** 1 Institute of Gastroenterology, Guangzhou University of Chinese Medicine, Guangzhou, Guangdong, China; 2 Department of Neurosurgery, the Second Affiliated Hospital of Soochow University, Suzhou, Jiangsu, China; University Hospital Llandough, UNITED KINGDOM

## Abstract

**Aim:**

This meta-analysis analyzed the efficacy and safety of traditional Chinese medicine (TCM) for the treatment of irritable bowel syndrome with constipation (IBS-C).

**Methods:**

We searched seven electronic databases for randomized controlled trials investigating the efficacy of TCM in the treatment of IBS-C. The search period was from inception to June 1, 2017. Eligible RCTs compared TCM with cisapride and mosapride. Article quality was evaluated with the Cochrane Risk Bias Tool in the Cochrane Handbook by two independent reviewers. Begg’s test was performed to evaluate publication bias. Review Manager 5.3 and Stata 12.0 were used for analyses.

**Results:**

Eleven eligible studies comprising a total of 906 participants were identified. In the primary outcome, TCM showed significant improvement in overall clinical efficacy compared with cisapride and mosapride (odds ratio [OR] = 4.00; 95% confidence interval [CI]: 2.74,5.84; P < 0.00001). In terms of secondary outcomes, TCM significantly alleviated abdominal pain (OR = 5.69; 95% CI: 2.35, 13.78; P = 0.0001), defecation frequency (OR = 4.38; 95% CI: 1.93, 9.93. P = 0.0004), and stool form (OR = 4.96; 95% CI: 2.11, 11.65; P = 0.0002) in the treatment group as compared to the control group. A lower recurrence rate was associated with TCM as compared to cisapride and mosapride (OR = 0.15; 95% CI: 0.08, 0.27; P < 0.00001). No adverse effects were observed during TCM treatment.

**Conclusions:**

TCM showed greater improvement in terms of clinical efficacy in the treatment of IBS-C than cisapride and mosapride, although it was not possible to draw a definitive conclusion due to the small sample size, high risk, and low quality of the studies. Large multi-center and long-term high-quality randomized control trials are needed.

## 1. Introduction

Irritable bowel syndrome (IBS) is one of the most common functional bowel disorders, which is characterized by recurrent abdominal pain or discomfort with accompanying disturbances in defecation that cannot be attributed to any demonstrable biochemical, structural, or anatomical abnormality [1, 2]. Thus, the diagnosis of IBS is based on symptoms, according to which the disorder is classified as IBS-C (IBS with pain or discomfort and predominant constipation), IBS-D (IBS with diarrhea), IBS-M (mixed IBS), or IBS-U (unsubtyped) [3, 4]. Epidemiologic studies have reported a high prevalence of IBS ranging from 5%–22% in various countries [5, 6], 2.9%–15.6% in Asian countries [7], and 0.82%–11.5% in China [8, 9]. Although IBS causes no physical injury to patients, it reduces their quality of life and consumes a significant amount of medical resources due to its chronicity and frequency of symptoms [10].

Drugs that promote gastrointestinal motility are the main treatment for IBS-C; for example, the 5-hydroxytryptamine (serotonin; 5-HT) agonists cisapride and mosapride provide effective relief of the predominant symptoms. However, long-term use of these drugs is associated with a risk of cardiovascular events [11]. Many IBS patients seek alternative treatments such as traditional Chinese medicine (TCM) [12–14], in which treatment is based on syndrome differentiation. TCM represent one aspect of Chinese medical philosophy that is characterized by its emphasis on maintaining and restoring balance[15].

A series of systematic reviews evaluating the effectiveness of TCM for treating IBS-D treatment have been published [16–20]. However, there is little information on the efficacy of TCM for the treatment of IBS-C. To address this issue, we carried out a meta-analysis to evaluate the safety and efficacy of TCM for IBS-C treatment.

## 2. Methods

### 2.1. Data sources and search strategy

Seven electronic databases including PubMed, Springer, EMBASE, CNKI(China National Knowledge Infrastructure), CBM (Chinese Biomedicine Database), WanFang, and VIP (Chinese Scientific Journals Database) were searched for randomized controlled trials (RCTs) evaluating the effects of TCM and gastrointestinal motility drugs on the clinical outcome of IBS-C. The search period was from inception to June 1, 2017. The following key words were used for our search: “Traditional Chinese medicine”, “irritable bowel syndrome”, “IBS”, “Constipation Type of Irritable Bowel Syndrome”, “IBS-C”, “clinical research”, and “clinical trial”.

### 2.2. Selection criteria

Two reviewers (D.-Y.L. and Y.-K.D.) independently reviewed the full-text versions of all the articles retrieved in the literature search to identify eligible studies. Conflicts in study selection were resolved by a third reviewer (J.-O.Y). Inclusion criteria were as follows: (1) IBS-C was definitively diagnosed according to Rome II[21], III[22], or IV criteria[23]; (2) clinical trial; (3) treatment groups used only traditional Chinese medicine (decoction, granules, capsule or tablet), while control groups used mosapride or cisapride (capsule or tablet); (4) randomized controlled trial (RCT); (5) duration of the treatment was at least 4 weeks for all groups; and (6) published in any language. Exclusion criteria were as follows: (1) not IBS-C; (2) IBS-D, IBS-M, IBS-U; (3) duplicate publication; (4) included subjects with severe enteric disease or who developed a malignancy, heart failure, or renal failure during the study period, and (5) publication was a commentary, editorial, reviews or case report.

### 2.3 Efficacy evaluation index

The indicator of primary outcome was overall clinical efficacy; secondary outcome indicators were symptom improvement rate, recurrence rate, and adverse reaction. According to the TCM Illness Diagnosis Affect Standard and Guidance Principle of Clinical Study on New Drug of Traditional Chinese Medicine [24, 25], efficacy evaluation was divided into four grades: cured, markedly effective, effective, and ineffective. The nimodipine method [24] was used to assess changes in the efficacy index, which was calculated with the formula [(pre-treatment symptom score − symptom score after treatment / pre-treatment symptom score)] × 100%.

### 2.4 Literature quality evaluation

The Cochrane Risk Bias Tool in the Cochrane Handbook for Systematic Reviews of Interventions was used to assess the quality of included studies, which supplemented by Jadad score[26]. Evaluation of methodological quality was also assessed by two independent reviewers, and a third reviewer was consulted when there were discrepancies.

### 2.5. Data extraction

Two reviewers (D.-Y.L. and Y.-K.D.) used a standardized data extraction form to independently extract data from studies that met the inclusion criteria, including (1) general information, including name of the first author, publication year, sample size, age, disease duration, trial duration, and intervention; (2) details of the study design, including descriptions of randomization methods, blinding, allocation concealment, and bias prevention; (3) clinical outcomes at the end of the treatment, number of withdrawals or dropouts, and follow-up periods; and (4) adverse events during treatment. Extracted data were cross-checked by the two reviewers. In cases of uncertainty about eligibility, a third reviewer was consulted.

### 2.6. Statistical analysis

Outcomes for which data could not be compared directly across studies were synthesized qualitatively, as were outcomes for which insufficient data were reported across studies. Outcomes for which sufficient, equivalent data were reported across studies were meta -analyzed using Forest plots generated with RevMan 5.3[27]. Enumerated data were analyzed using OR or relative risk (RR) and 95% CI. The χ^2^ test and the inconsistency index statistic (I^2^) for heterogeneity were conducted [28]. If substantial heterogeneity existed (I^2^> 50% or P< 0.05), a random effect model was applied. If there was no observed heterogeneity, fixed effect models were chosen [29]. A sensitivity analysis was done to explore potential sources of heterogeneity. Begg’s test was performed to evaluate publication bias [30]. Review Manager 5.3 and Stata 12.0 were used for analyses.

## 3. Results

### 3.1 Study description

**Fig 1:**
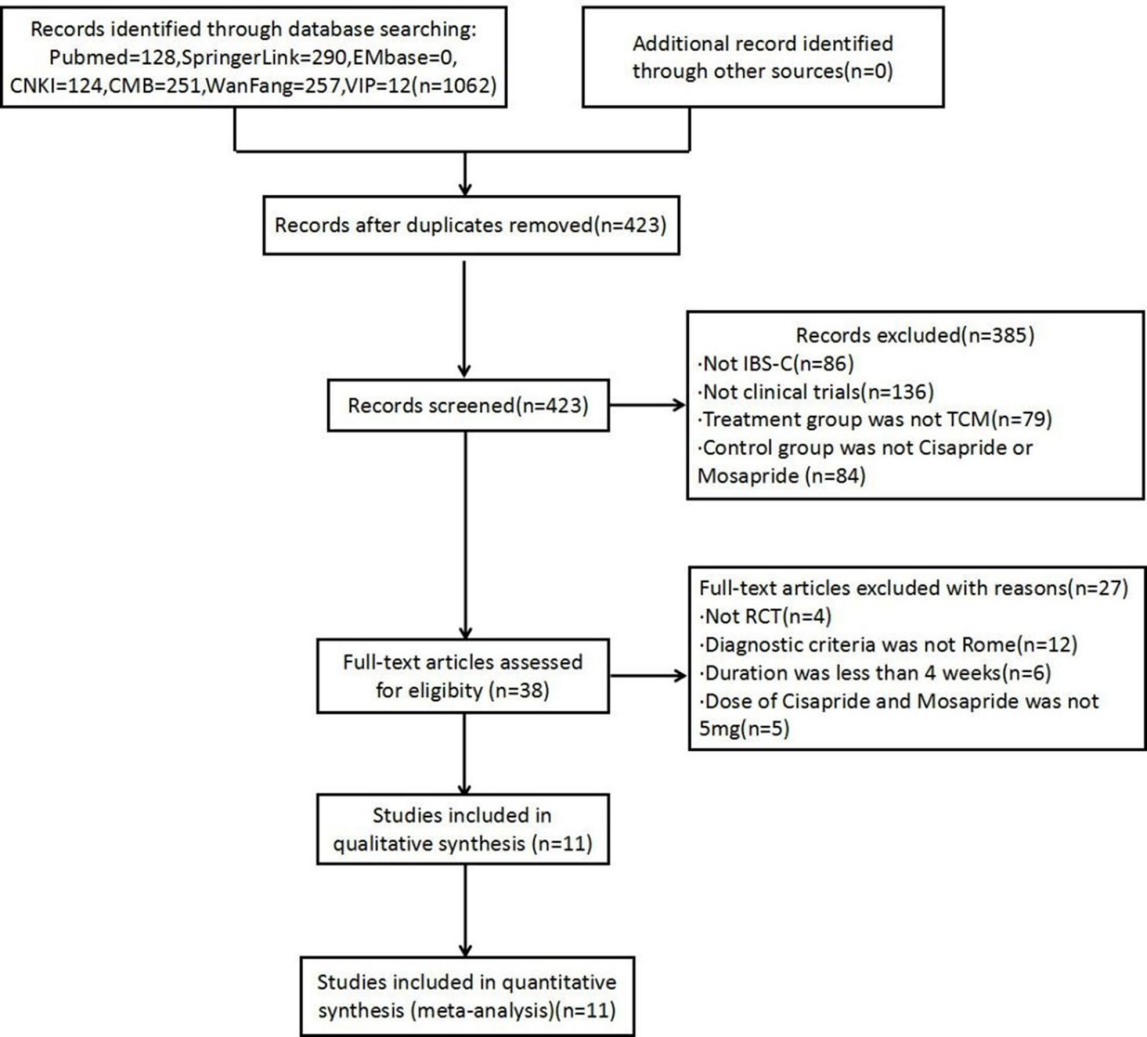
Flow diagram of literature search.

The literature search identified 1062 studies. The detailed screening process is shown in Fig 1(**Fig 1** Flow diagram of literature search). Based on the inclusion and exclusion criteria, 11 RCTs [31–41] were ultimately included in the meta-analysis, comprising 906 participants (494 in the trial group and 412 in the control group) ranging in age from 17 to 72 years old; in two studies [32, 40] the participants’ ages were not mentioned. Disease duration was between 3 months and 12 years; three articles [35, 36, 38] did not mention the duration. The course of treatment was between 4 and 8 weeks. Ten of the treatment groups used a Chinese medicine decoction which refers to the liquid formulation that is obtained by boiling pieces or coarse grains of herbs in water or soaking the crude medicine in boiling water followed by filtration [42].while one used granules [35]. In the control group, four articles used cisapride [31–34] and seven articles used mosapride [35–41]. All selected RCTs reported overall clinical efficacy at the end of the treatment, and three [31–33] reported recurrence rate and follow-up periods. The characteristics of the studies are listed in Table 1.

**Table 1:** Characteristics of the studies included in the meta-analysis.

First Author, Year	Country	Diagnostic Criteria	N	Age (years)	Disease duration (years)			Duration (weeks)	Intervention		Follow-up	Recurrence rate
			(T vs. C)		T	C			T	C		
Cai 2005 (31)	China	Rome II	60 vs. 30	19–72	0.25–17	0.42–6.5		4	Decoction of liver -soothing and stomach -regulating, 200mL, b.i.d	Cisapride tablets, 5mg, tid	6m	mentioned
Chen 2005 (32)	China	Rome II	41 vs. 37	Not mentioned	1.55±0.45	1.49±0.51		4	Yiji II recipe, t.i.d	Cisapride tablets, 5mg, tid	4w	mentioned
Wu 2006 (33)	China	Rome II	85 vs. 85	26–70	1–10	1–9.5		8	Decoction of Buqi Jianpi, 150mL, b.i.d	Cisapride tablets, 5mg, tid	4w	mentioned
Wang 2008 (34)	China	Rome II	50 vs. 33	20–65	4.9±1.54	4.5±1.84		4	Yangxue Tongfu Decoction, 300mL,b.i.d	Cisapride tablets, 5mg, tid	N.R	N.R
Li 2011 (35)	China	Rome III	50 vs. 35	48.01±6.70	3.26±1.33			4	Compound granules with Regulating Qi and Moistening the Intestine -s, 100mL, b.i.d	Mosapride citrate tablets, 5mg, t.i.d	N.R	N.R
Liao 2011 (36)	China	Rome III	35 vs. 20	T:51.7±12.9 C:52.6±12.9	N.R		N.R	4	Modified Sini Powder and Liumo Decoction, b.i.d	Mosapride citrate tablets, 5mg, t.i.d	N.R	N.R
Qian 2011 (37)	China	Rome III	25 vs. 25	21–61	2.51±1.23		2.8±1.18	4	Modified Fluid-increasing Decoction, b.i.d	Mosapride citr -ate tablets, 5mg, t.i.d	N.R	N.R
Chen 2013 (38)	China	Rome III	20 vs. 20	T:41.89±5.67 C:40.31±6.98	N.R		N.R	4	Decoction of Replenishing Qi to invigorate the Spleen and ventilating the Lung,200mL, b.i.d	Mosapride tablets, 5mg, t.i.d	N.R	N.R
Zhang 2014 (39)	China	Rome III	48 vs. 48	22–64	1–13		1–12	4	Modified Guipi Decoction and Xiaoyao Powder, b.i.d	Mosapride capsules, 5mg, t.i.d	N.R	N.R
Wang 2015 (40)	China	Rome III	40 vs. 39	Not mentioned	0.25–11		0.5–12	8	Modified Sanzang Tiaohe Runchang Decoction, 150 mL, b.i.d	Mosapride citrate tablets, 5mg, t.i.d	N.R	N.R
Zhang 2016 (41)	China	Rome III	40 vs. 40	21–68	4.3±1.2		4.5±1.5	4	Treatment base on TCM Syndrome Differentiation, b.i.d	Mosapride citrate tablets, 5mg, t.i.d	N.R	N.R

N.R = not reported; T = Treatment group; C = control group; b.i.d = bisindie; t.i.d = terindie;VS. = Versus.

### 3.2 Methodological quality

**Fig 2:**
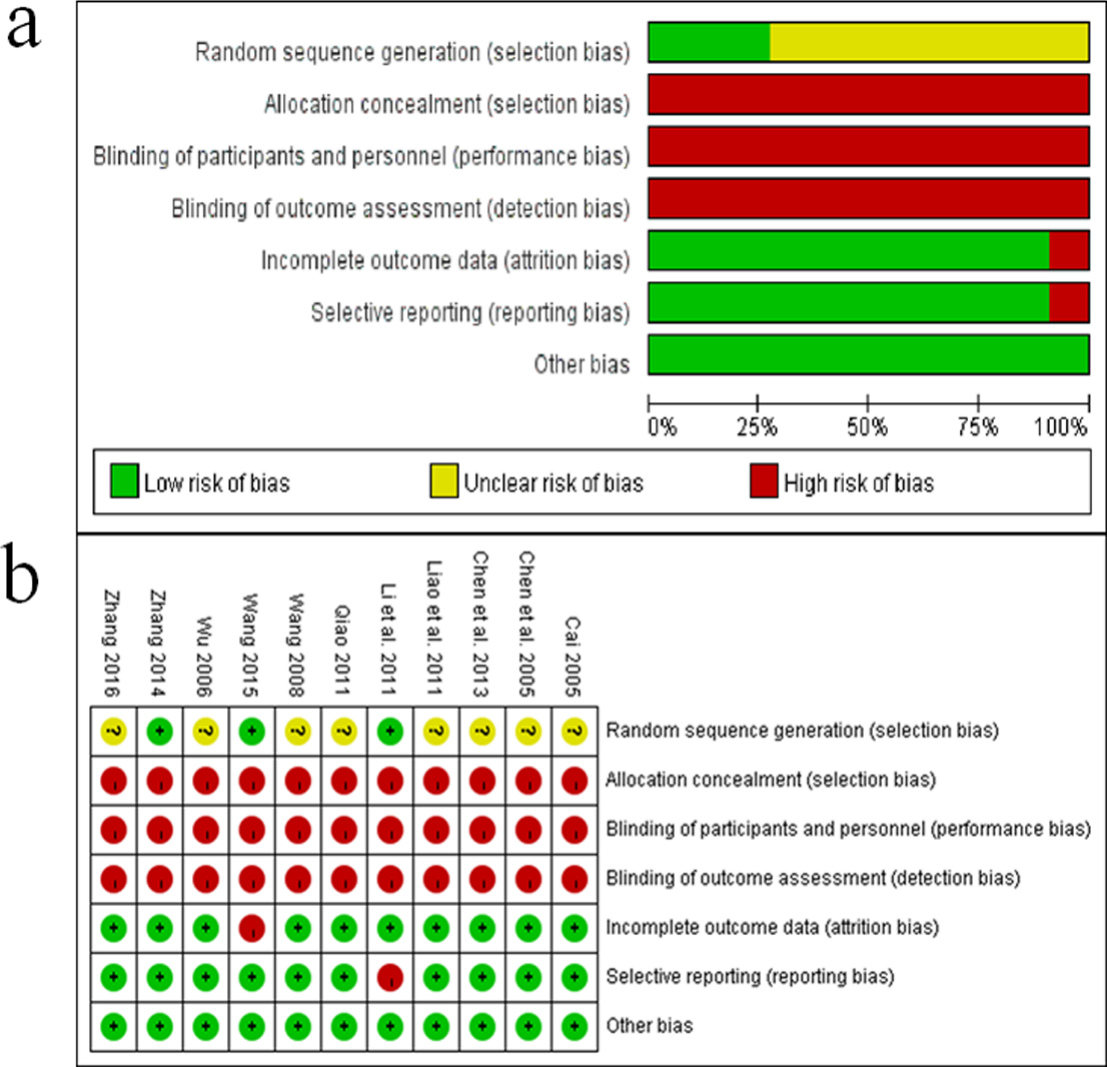
Methodological quality assessment of the risk of bias.

All included studies were randomized studies with comparable baselines. Two studies [35, 39] used random number tables: one [40] according to the principle of minimum distribution of unbalanced index, and one [32] as a simple random table. The remaining seven studies did not mention a specific method. Ten studies did not mention double blinding or allocation concealment; only one [35] mentioned the use of sealed envelopes although a non-blinded approach was adopted. One trial [40] reported patient dropout, but an Intention-to-Treat (ITT) analysis was not performed. For other types of bias, seven studies [32, 35–37, 39–41] were rated as unclear risk due to the lack of information on age, disease duration, and dosage of Chinese medicine. This meta-analysis was considered as having a high risk of bias (Fig 2 Methodological quality assessment of the risk of bias).A description of the evaluation of methodological quality of the 11 trials can be found in Table 2.

**Table 2:** Evaluation of methodological quality of the included studies.

Study ID	Baseline	Randomization	Double Blinding	Withdrawal or dropout	Allocation concealment	Side effects	Jadad scores
Cai 2005 (31)	Comparability	Mentioned not described	N.R	N.R	N.R	N.R	1
Chen2005 (32)	Comparability	Simple random table	N.R	N.R	N.R	C:6 cases	1
Wu 2006 (33)	Comparability	Mentioned not described	N.R	N.R	N.R	No	1
Wang2008(34)	Comparability	Mentioned not described	N.R	N.R	N.R	N.R	1
Li 2011(35)	Comparability	Random number table	Unblinded	N.R	Mentioned not described	N.R	2
Liao 2011 (36)	Comparability	Mentioned not described	N.R	N.R	N.R	N.R	1
Qian 2011 (37)	Comparability	Mentioned not described	N.R	N.R	N.R	No	1
Chen 2013(38)	Comparability	Mentioned not described	N.R	N.R	N.R	N.R	1
Zhang2014(39)	Comparability	Random number table	N.R	N.R	N.R	N.R	2
Wang 2015(40)	Comparability	The principle of minimum distribution of unbalanced index	N.R	C:1 case	N.R	C:1 case	3
Zhang2016 (41)	Comparability	Mentioned not described	N.R	N.R	N.R	N.R	1

N.R = not reported; C = control group.

### 3.3 Primary outcome: Overall clinical efficacy

**Fig 3:**
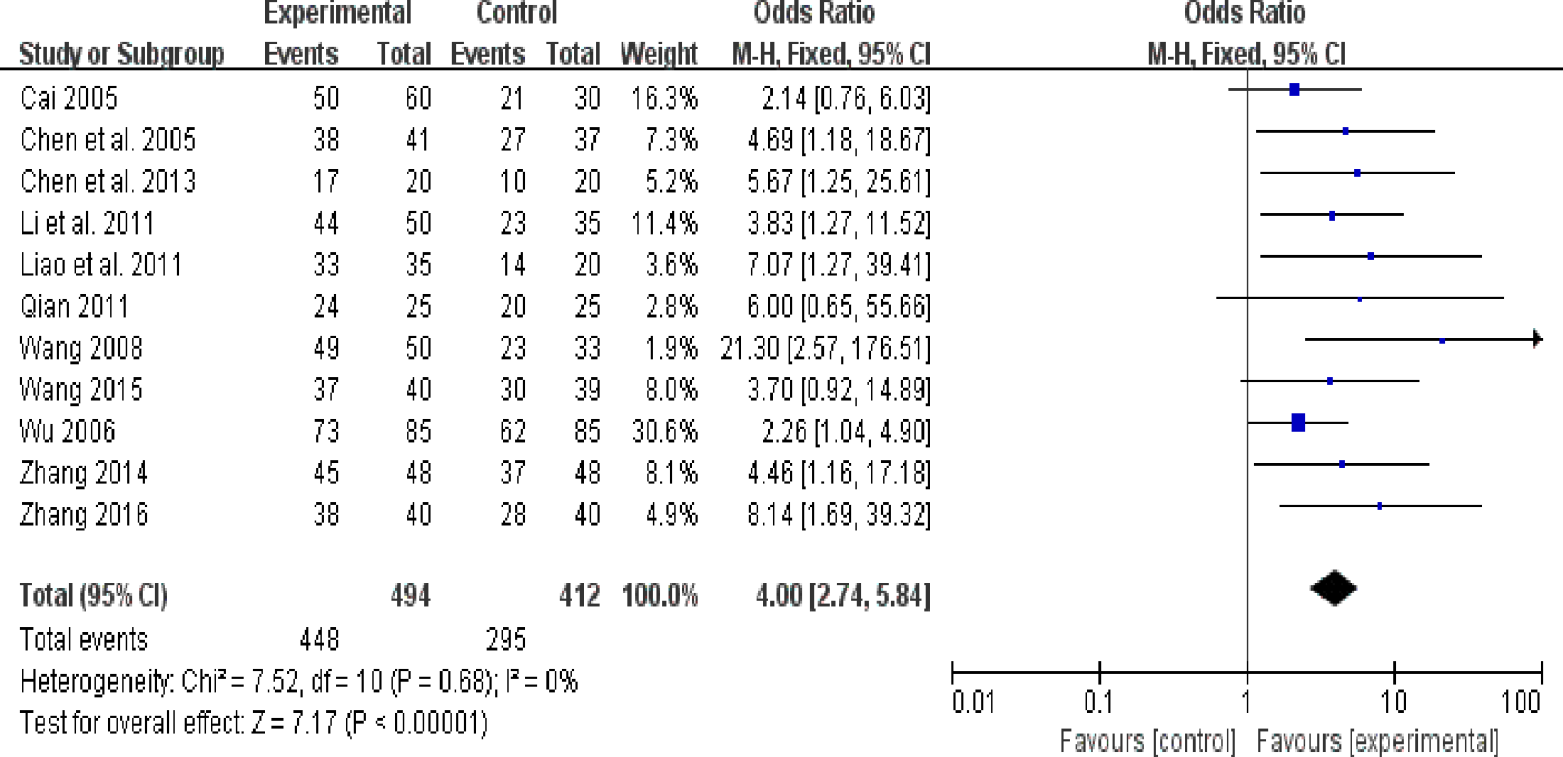
Forest plot of primary outcomes (fixed effect model).

**Fig 4:**
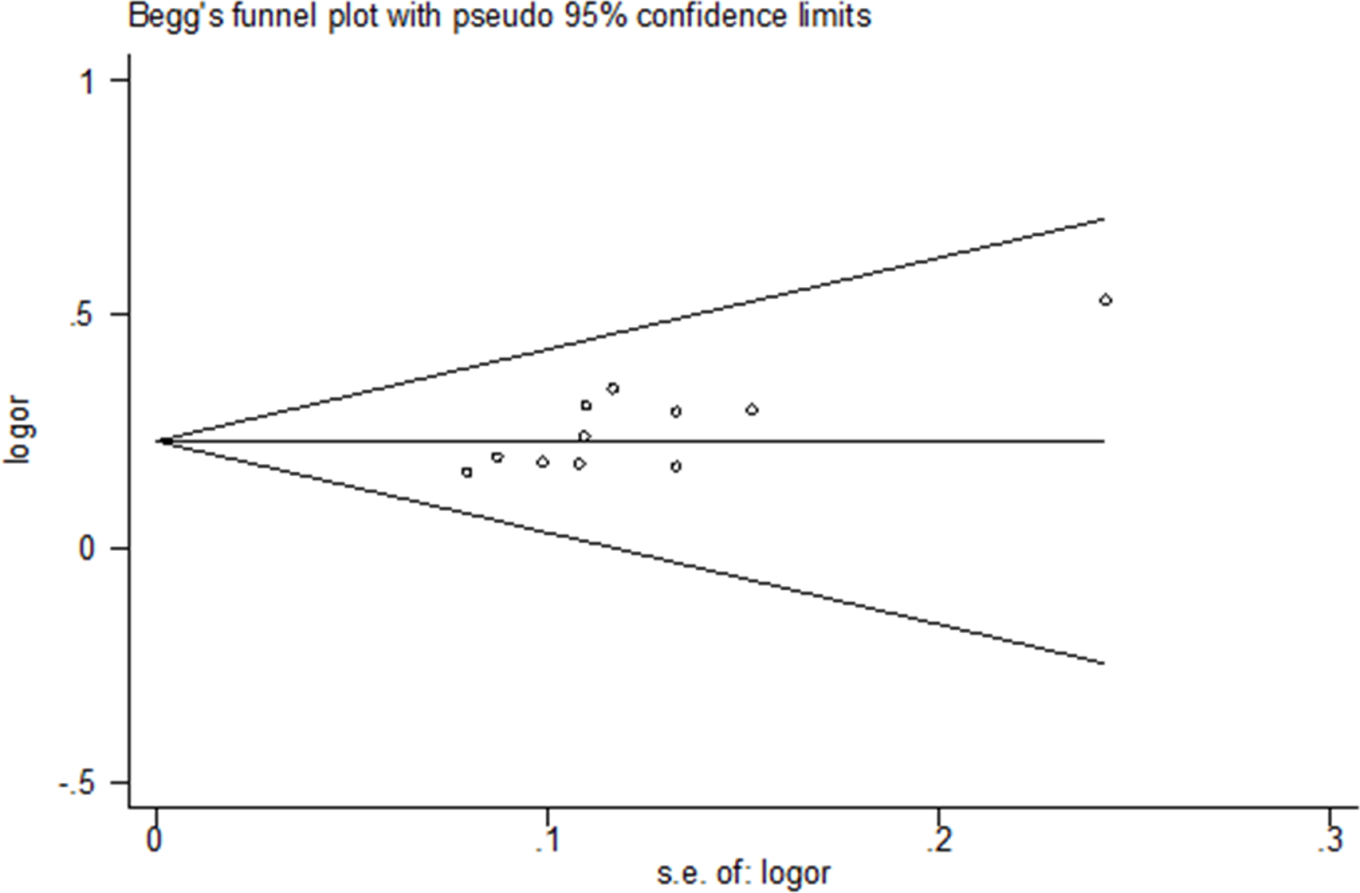
Begg’s funnel plot analysis of primary outcomes.

Overall clinical efficacy was evaluated in 11 trials. The study included 906 patients; 494 were assigned to treatment groups, whereas 412 were assigned to control groups. TCM significantly improved overall clinical efficacy as compared to cisapride and mosapride (OR = 4.00; 95% CI: 2.74, 5.84; P < 0.00001) (Fig 3 Forest plot of primary outcomes (fixed effect model). No heterogeneity was observed (χ^2^ = 7.52, P = 0.68, I^2^ = 0%). Funnel plot analysis demonstrated evidence of publication bias (Begg’s test, P = 0.013) (Fig 4 Begg’s funnel plot analysis of primary outcomes).

### 3.4 Secondary outcomes

#### Abdominal pain

**Fig 5:**
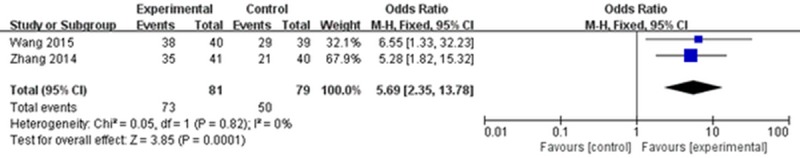
Forest plot of improvements in abdominal pain (fixed effects model).

Five of the 11 studies [32, 35, 36, 39, 40] reported the outcome of abdominal pain improvement; three of these [32, 35, 36] used symptom scores, although the scoring criteria were inconsistent. The remaining two studies [39, 40], in which evaluation was based on efficacy, were incorporated into the meta-analysis. TCM resulted in greater abdominal pain alleviation relative to controls (OR = 5.69; 95% CI: 2.35, 13.78; P = 0.0001), with no statistical heterogeneity (χ^2^ = 0.05, P = 0.82, I^2^ = 0%) (Fig 5 Forest plot of improvements in abdominal pain (fixed effects model)).

#### Defecation frequency

**Fig 6:**
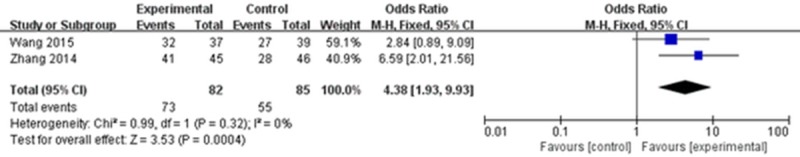
Forest plot of the improvement in defecation frequency (fixed effects model).

Three studies [32, 39, 40] reported an alleviation in defecation frequency. One [32] used symptom scores to assess the alleviation whereas the other two [39, 40] evaluated the efficacy and were therefore included in our analysis. TCM significantly alleviated defecation frequency as compared to control groups (OR = 4.38; 95% CI: 1.93, 9.93. P = 0.0004). There was no apparent heterogeneity (χ^2^ = 0.99, P = 0.32, I^2^ = 0%) (Fig 6 Forest plot of the improvement in defecation frequency (fixed effects model)).

#### Stool form

**Fig 7:**
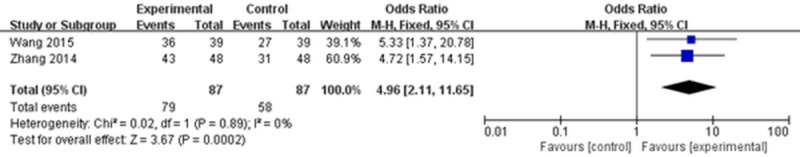
Forest plot of the improvement in stool form (fixed effects model).

Four studies [32, 36, 39, 40] reported an alleviation in stool form according to symptoms. Two [39, 40] used the same criteria and were included in the analysis. TCM alleviated stool form relative to the control group (OR = 4.96; 95% CI: 2.11, 11.65; P = 0.0002); there was no heterogeneity observed (χ^2^ = 0.02, P = 0.89, I^2^ = 0%) (Fig 7 Forest plot of the improvement in stool form (fixed effects model)). There were limited descriptions in the literature of other symptoms such as abnormal bowel movements, abdominal tenderness [32], incomplete evacuation, fatigue [36] or anorexia [40]; this information was therefore only useful for a qualitative analysis.

### 3.5 Recurrence rate and adverse events

**Fig 8:**
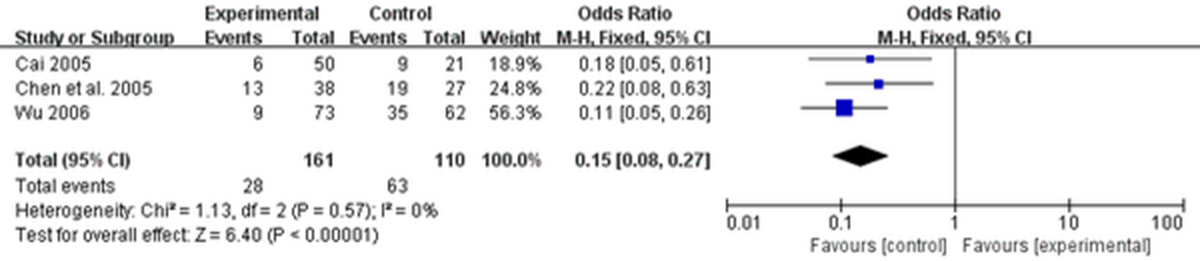
Forest plot of recurrence rate (fixed effects model).

Among included studies, three trials [31–33] reported recurrence rate during a 6-month follow-up after the treatment course. The fixed-effects model showed that TCM had a significantly lower recurrence rate than cisapride and mosapride (OR = 0.15; 95% CI: 0.08, 0.27; P < 0.00001). There was no observed heterogeneity (I^2^ = 0%, P = 0.57) (Fig 8 Forest plot of recurrence rate (fixed effects model)). Summary of meta-analysis outcomes can be found in Table 3.

**Table 3:** Summary of meta-analysis outcomes.

Outcomes	N	Patients (T/C)	Overall effect		Heterogeneity	
			OR (95% CI)	P	I^2^	P
Primary outcome						
Overall clinical efficacy	11	494/412	4.00[2.74,5.84]	<0.0001	7.52	0.68
Secondary outcomes						
Abdominal pain	2	81/79	5.69[2.35,13.78]	0.0001	0.05	0.82
Defecation frequency	2	82/85	4.38[1.93,9.93]	0.0004	0.99	0.32
Stool form	2	87/87	4.96[1.37,20.78]	0.0002	0.02	0.89
Recurrence rate	3	161/110	0.15[0.08,0.27]	<0.0001	1.13	0.57

N: number of studies; OR: odds ratio; CI:confidence interval; T = Treatment group; C = control group.

Two studies [32, 40] mentioned adverse events in the control group. One patient withdrew from the study after reporting dizziness and dry mouth [40], and two cases of low abdominal pain, three cases of mild diarrhea, and one case of bowel movements were also reported [32]. No adverse effects were observed during TCM treatment.

## Discussion

This meta-analysis investigated the efficacy of TCM in the treatment of IBS-C as compared to gastrointestinal motility drugs. The results showed that TCM produced greater alleviation in IBS-C symptoms than cisapride and mosapride. Data from only two trials were included in the analysis of secondary outcomes, and the analysis showed that TCM alleviated abdominal pain (OR = 5.69), defecation frequency (OR = 4.38) and stool form (OR = 4.96) as compared to the control group. Furthermore, patients treated with TCM had a significantly lower recurrence rate than controls (OR = 0.15), with no adverse events. For instance, in one study [35] patients were aware of which treatment they received, whereas another study [31] excluded one patient due to adverse effects but an ITT analysis was not performed, potentially leading to incomplete outcome data. Therefore, although TCM appears to be superior to gastrointestinal motility drugs for the treatment of IBS-C, the efficacy of traditional Chinese medicine requires further study.

The pathophysiology of IBS is not fully understood; it is thought to involve many factors, including gastrointestinal dysmotility [43], visceral hypersensitivity [44, 45], intestinal mucosa activation [4], increased intestinal permeability [46], inflammation, epigenetics, and genetics [47]. Many studies have reported that TCM is effective for IBS-C treatment. Ma Zhi Jiang Zhuo was shown to relieve constipation symptoms in a rat model of IBS-C, possibly by increasing the rate of small intestine propulsion and reducing 5-HT level in the colon [48]. Maziren pills increased visceral hypersensitivity by decreasing 5-HT levels in IBS-C rats [49]. Clinical trials have shown that flavored Maren pills improved abdominal symptoms and constipation in IBS-C by modulating the balance between pro- and anti-inflammatory cytokines—i.e., by decreasing interleukin (IL)-6 and increasing IL-10 levels [50]. On the other hand, clinical data have shown that liver-discharging, qi-regulating, and intestine-moistening therapies can relieve abdominal pain and improve constipation symptoms, possibly by reducing plasma vasoactive intestinal peptide (VIP) and somatostatin (SS) levels [51]. Kuanzhongjieyutang can improve IBS symptoms and patients’ quality of life by reducing plasma levels of VIP, SS, and 5-HT and modulating the brain-gut axis, thereby decreasing visceral hypersensitivity and improving intestinal motor function [52]. Ballast capsules can improve IBS symptoms by increasing motilin and decreasing endothelin levels [53]. Additional studies of these herbal formulae are needed in order to clarify the mechanisms underlying these effects.

There were several limitations to this meta-analysis. First, the course of treatment was between 4 and 8 weeks, and three trials [31–33] reported a 6-month follow-up, which was not sufficiently long to evaluate the long-term efficacy of TCM for IBS-C in all included studies. Second, only three studies [33, 37, 39] reported safety evaluation. In one, routine blood, urine, and stool tests were performed and liver and kidney function were evaluated before and after treatment [33]. In the other two studies, enteroscopy [37] and electrocardiography [39] were performed in addition to these tests. Results from all of the tests were normal. The safety of TCM must be confirmed in future studies. Third, although the results showed that disease recurrence rate in patients treated by TCM was relatively low, this may be unreliable due to the limited number of studies that were suitable for analysis [31–33]. Fourth, dropouts were reported in one study [40], but ITT analysis was not performed, which could lead to bias in evaluating the clinical efficacy of the intervention. Finally, our meta-analysis included published studies that were conducted in China and published in Chinese, only, and some of the unpublished data were not available for various reasons. In general, the studies had a high risk of bias and low methodological quality. Whether the effect size of TCM would be similar in large-scale trials remains to be determined.

## Conclusions

IBM-C patients treated with TCM showed greater improvement than those treated with cisapride or mosapride. However, it is difficult to draw definitive conclusions from our meta-analysis due to the small sample size, high heterogeneity, and low quality of the included studies. Large, multi-center, long-term, and high-quality RCTs are needed to confirm whether TCM is a viable alternative to conventional drugs for the treatment of IBS-C.

## Supporting information

S1 File A sample search strategy. (DOCX)

S2 File PRISMA 2009 checklist. (DOCX)
