# Supplementary material for: Systematic review and meta-analysis of traditional Chinese medicine in the treatment of constipation-predominant irritable bowel syndrome
Source: PLoS One. 2017 Dec 18;12(12):e0189491. doi: 10.1371/journal.pone.0189491 (PMC5734785; doi:10.1371/journal.pone.0189491)
Supplement: S1 File — (DOCX) [file pone.0189491.s001.docx]

**A sample search strategy**

We comprehensively searched for publications in PubMed database from its inception through June 1, 2017: (“irritable bowel syndrome OR IBS OR Constipation Type of Irritable Bowel Syndrome OR IBS-C”) AND (“traditional Chinese medicine”) .
